# Editorial: Integrative Physiology of Common Chronic Musculoskeletal Disorders

**DOI:** 10.3389/fphys.2022.971103

**Published:** 2022-07-11

**Authors:** Brian C. Clark, Dustin R. Grooms, Timothy Etheridge, Daniel J. Wilkinson, Shouan Zhu, W. David Arnold, Nathaniel J. Szewczyk

**Affiliations:** ^1^ Ohio Musculoskeletal and Neurological Institute (OMNI), Ohio University, Athens, OH, United States; ^2^ Department of Biomedical Sciences, Ohio University, Athens, OH, United States; ^3^ School of Rehabilitation and Communication Sciences, Ohio University, Athens, OH, United States; ^4^ Department of Sport and Health Sciences, College of Life and Environmental Sciences, University of Exeter, Exeter, United Kingdom; ^5^ MRC-ARUK Centre for Musculoskeletal Aging Research and National Institute of Health Research, Biomedical Research Centre, Royal Derby Hospital Centre, School of Medicine, University of Nottingham, Derby, United Kingdom; ^6^ Department of Neurology, Ohio State University, Columbus, OH, United States

**Keywords:** pain, injury, fracture, ACL, muscular dystrophy, muscle, disuse, cachexia

Musculoskeletal (MSK) disorders are most one of the most commonly reported medical conditions (Figure 1), and are the leading cause of disability in the U.S. as they account for more than half of chronic conditions in people over age 50 (Figure 1) (Initiative USBaJ, 2020). The costs dwarf those of other conditions, coming close to 6% of the U.S. gross domestic product (Initiative USBaJ, 2020). Arthritis, pain, and trauma, as well as sarcopenia, cancer cachexia and other skeletal muscle diseases are key conditions that drive this societal burden (Arthur et al., 2014; Goates et al., 2019; Initiative USBaJ, 2020). These conditions are associated with a myriad of complex physiological changes not only involving the MSK system, but also the nervous system through integrated feedback and feedforward processes. Accordingly, we hosted a special research topic issue for *Frontiers in Physiology* that focused on integrative physiology of common chronic MSK disorders. We received 13 submissions for this special topic issue, of which seven were judged by us (the editors) and the peer-reviewers as meritorious for inclusion. These are briefly summarized here.

**FIGURE 1:**
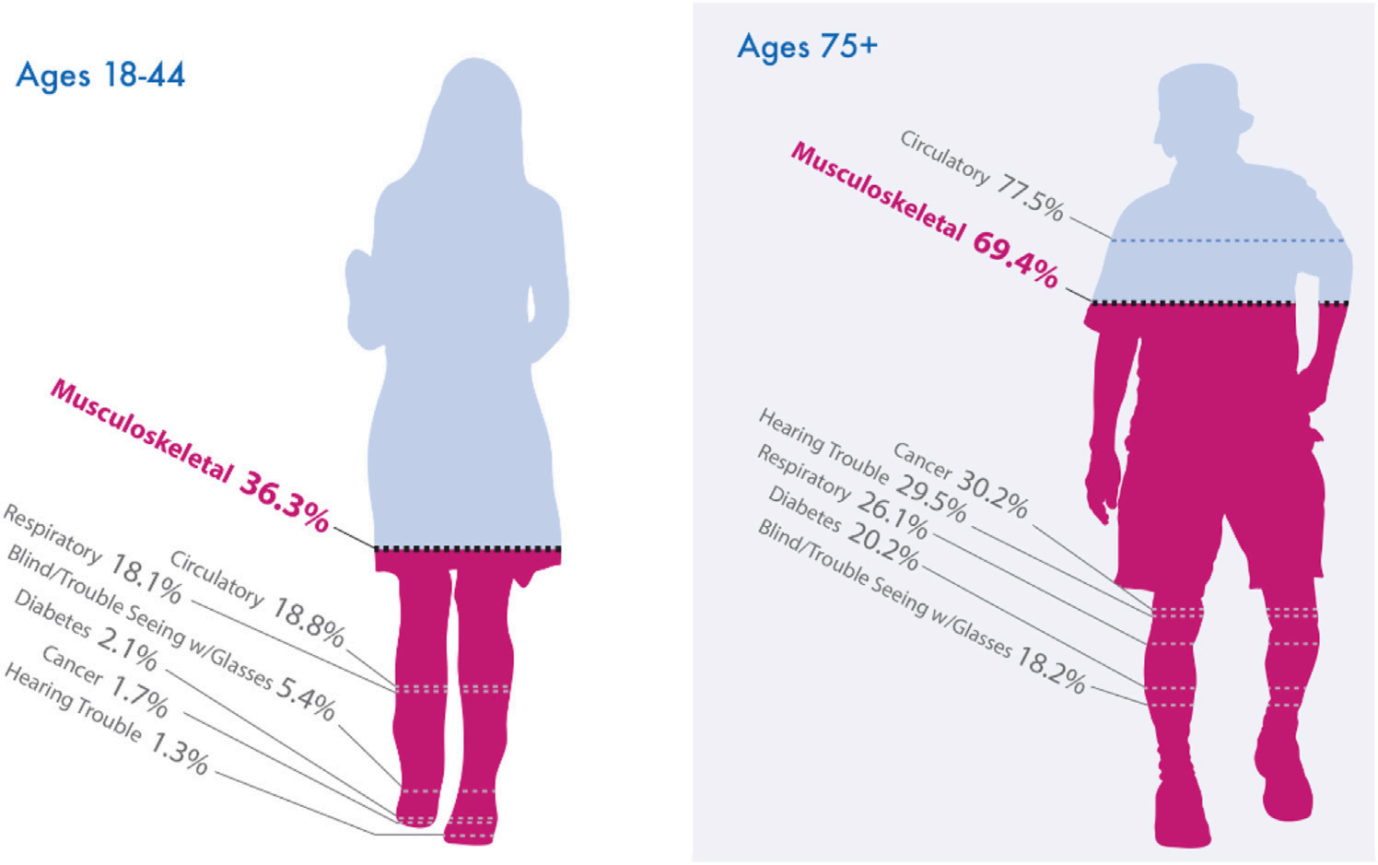
Musculoskeletal (MSK) disorders are most one of the most reported medical conditions. Not surprisingly, as people age, conditions such as hearing and vision problems, cancer, and heart disease become more common. Still, across all adult age categories, musculoskeletal conditions are either the most commonly reported medical conditions (among those under 65) or second most commonly reported (among those 65 and older). Figure from The Burden of Musculoskeletal Diseases in the United States - United States Bone and Joint Initiative, 2020.

## Injury and Pain

Bird and others (Bird et al.) identified movement strategies associated with the development of MSK injuries in military personnel, and observed that lower braking rate, braking impulse, and propulsive impulse during a countermovement jump were associated with increased MSK injury risk. Additionally, higher degrees of flexion and longer countermovement jump phase durations were linked to increased MSK injury risk. Interesting, males were distributed equally across injury clusters while females were primarily distributed in the high-risk cluster. Their findings hold strong clinical relevance to numerous fields as they provide actionable thresholds that can be applied to screening measures to identify individuals at increased MSK injury risk.

Using a similar design, Koltun and others (Koltun et al.) examined whether baseline measures of bone density, geometry, and estimated bone strength (assessed via peripheral quantitative computed tomography) are predictive of tibial bone stress injuries in military personnel. Their findings indicated that narrower bones, with reduced circumference, lower total area, and lower estimated bending strength were associated with increased risk for tibial bone stress injury during military training. These findings highlight the importance of assessing factors beyond bone mineral density to understand fracture risk.

Davi and others (Davi et al.) leveraged a non-invasive anterior cruciate ligament (ACL) injury rodent model to explore the effect of an isolated ACL injury on mitochondrial function, atrophy, and muscle phenotypic transitions. They observed that mitochondria-derived reactive oxygen species increased following ACL injury concomitant with a two-fold reduction in the mitochondrial respiratory control ratio. Male rats displayed myofiber atrophy and fiber type shifts while female rats displayed no such changes. A positive relationship was observed between mitochondrial respiration and myofiber size. Thus, they concluded that long-lasting impairments in mitochondrial health are present after ACL injury and play a key role in the dysregulation of quadriceps muscle size and composition.

## Skeletal Muscle Wasting and Weakness Disorders

Deschenes and others (Deschenes et al.) used a rat model to examine whether muscle unloading imposed during juvenile development would elicit more severe disruption in neuromuscular function than when imposed on mature neuromuscular systems. Juvenile and mature rats were divided into a 2-weeks hindlimb unloading group or a control group. The unloading protocol resulted in similar degrees of atrophy in juvenile and mature muscles. Interestingly, though, the juvenile muscle displayed significantly more loss of specific tension as well as more degradation in the function of the neuromuscular junction. These results indicate that juvenile neuromuscular systems are more sensitive to the effects of unloading than mature ones and suggest the primary locus of this developmental related difference is the neuromuscular junction.

Baumann and others (Baumann et al.) used wild-type and several dystrophic mouse models to determine if maintenance of plasmalemmal excitability during and after a bout of eccentric contractions is dependent on the dystrophin glycoprotein complexes rather than, solely, dystrophin expression. The contractions caused all mouse lines to lose torque with the deficits being greatest in the dystrophic mice. Notably, loss of torque did not correspond to a reduction in plasmalemmal excitability in wild type mice, but a more than 50% reduction in plasmalemmal excitability was observed in the dystrophic mice. An large amount of the variability (98%) of the torque loss was explained by reduction in plasmalemmal excitability in the dystrophic mice. Thus, by comparing mouse lines that had varying amounts and functionality of dystrophin and other dystrophin glycoprotein complexes they demonstrate the significant role the dystrophin glycoprotein complexes play in maintaining plasmalemmal excitability.

Dillon and others (Dillon et al.) probed the skeletal muscle proteome of cancer patients who were in a randomized trial investigating the effect of adjunct testosterone on body composition in late stage or recurrent head and neck or cervical cancer. Muscle biopsies were obtained before and after 7-weeks of treatment. Proteoforms showing significant changes were identified by mass spectroscopy, and lists of altered proteins were subjected to Gene Set Enrichment Analysis to identify affected pathways. Their findings indicated that cancer and standard of care treatment significantly altered the skeletal muscle proteome in a manner suggestive of loss of structural integrity, reduced contractile function, and disrupted metabolism. Proteomic analysis suggests that the addition of adjunct testosterone minimized the structural and contractile influence of cancer and its associated therapies.

Lastly, Young and others (Young et al.) describe and discuss the MSK effects of altered growth hormone action. Growth hormone, a peptide hormone that can signal directly through its receptor or indirectly through insulin-like growth factor 1, draws its name from its anabolic effects on muscle and bone but also has distinct metabolic effects in multiple tissues. In addition to its metabolic and MSK effects, growth hormone is closely associated with aging, with levels declining as individuals age. However, growth hormone action has been negatively correlated with lifespan. This review article provides an overview of the MSK effects of growth hormone using data from both clinical and animal models.

Collectively, these articles address the mechanisms, effects, and consequences of injury and pain (e.g., arthritis, trauma) as well as muscle wasting and weakness disorders. Overall, they represent basic and applied physiology work with relevance to these highly prevalent and burdensome conditions.
